# Prof. Bartlett’s Essay on Edematous Laryngitis

**Published:** 1850-03

**Authors:** 


### PROFESSOR BARTLETT'S ESSAY ON EDEMATOUS LARYNGITIS.

This admirable essay cannot fail to arrest the attention of the reader, and it will well repay the most diligent study. It is clear, forcible, full and complete in its various details, and richly deserves the attention of practitioners.

The proprietors of this Journal have published this essay in pamphlet form, for the benefit of non-subscribers. The pamphlet may be obtained at the office of Messrs. Prentice & Weissinger.

